# FooDrugs: a comprehensive food–drug interactions database with text documents and transcriptional data

**DOI:** 10.1093/database/baad075

**Published:** 2023-11-09

**Authors:** Blanca Lacruz-Pleguezuelos, Oscar Piette, Marco Garranzo, David Pérez-Serrano, Jelena Milešević, Isabel Espinosa-Salinas, Ana Ramírez de Molina, Teresa Laguna, Enrique Carrillo de Santa Pau

**Affiliations:** Computational Biology Group, Precision Nutrition and Cancer Research Program, IMDEA Food Institute, CEI UAM+CSIC, Carretera de Cantoblanco, 8, Madrid 28049, Spain; Computational Biology Group, Precision Nutrition and Cancer Research Program, IMDEA Food Institute, CEI UAM+CSIC, Carretera de Cantoblanco, 8, Madrid 28049, Spain; Computational Biology Group, Precision Nutrition and Cancer Research Program, IMDEA Food Institute, CEI UAM+CSIC, Carretera de Cantoblanco, 8, Madrid 28049, Spain; Centre of Research Excellence in Nutrition and Metabolism, Institute for Medical Research, University of Belgrade, National Institute of the Republic of Serbia, Tadeuša Košćuška 1, PAK 104 201, Belgrade 11 158, Serbia; Capacity Development in Nutrition—CAPNUTRA, Trnska 3, Belgrade 11000, Serbia; GENYAL Platform on Nutrition and Health, IMDEA Food Institute, CEI UAM+CSIC, Carretera de Cantoblanco, 8, Madrid 28049, Spain; GENYAL Platform on Nutrition and Health, IMDEA Food Institute, CEI UAM+CSIC, Carretera de Cantoblanco, 8, Madrid 28049, Spain; Computational Biology Group, Precision Nutrition and Cancer Research Program, IMDEA Food Institute, CEI UAM+CSIC, Carretera de Cantoblanco, 8, Madrid 28049, Spain; Computational Biology Group, Precision Nutrition and Cancer Research Program, IMDEA Food Institute, CEI UAM+CSIC, Carretera de Cantoblanco, 8, Madrid 28049, Spain

## Abstract

Food–drug interactions (FDIs) occur when a food item alters the pharmacokinetics or pharmacodynamics of a drug. FDIs can be clinically relevant, as they can hamper or enhance the therapeutic effects of a drug and impact both their efficacy and their safety. However, knowledge of FDIs in clinical practice is limited. This is partially due to the lack of resources focused on FDIs. Here, we describe FooDrugs, a database that centralizes FDI knowledge retrieved from two different approaches: a natural processing language pipeline that extracts potential FDIs from scientific documents and clinical trials and a molecular similarity approach based on the comparison of gene expression alterations caused by foods and drugs. FooDrugs database stores a total of 3 430 062 potential FDIs, with 1 108 429 retrieved from scientific documents and 2 321 633 inferred from molecular data. This resource aims to provide researchers and clinicians with a centralized repository for potential FDI information that is free and easy to use.

**Database URL:**  https://zenodo.org/records/8192515

**Database DOI:**  https://doi.org/10.5281/zenodo.6638469

## Introduction

Food and health are intimately related. Consumers are now more aware of the importance of following a proper diet and are willing to change their dietary patterns in order to maintain or improve their health status (1). This has led to a growing consumption of products such as food supplements, functional foods or herbal medicinal products, which contain bioactive compounds that can affect human health (2, 3). This shift in the perceived function of food supplements towards pharmaceutical products coincides with an increase in non-communicable diseases such as heart disease or cancer (2, 4). There is a growing number of patients that follow chronic pharmacological treatments, in many cases requiring the simultaneous intake of more than one medication (5). In this regard, the consumption of nutritional supplements with an active role to treat or prevent non-communicable diseases has increased during the last few years, raising concerns over the risk of interactions and adverse reactions between food items and pharmaceutical products (6).

Food–drug interactions (FDIs) are the consequence of a physical, chemical or physiologic relationship between a drug and either a product consumed as food or a dietary supplement (7). These interactions are frequent in orally administered drugs and can affect both their pharmacodynamics and their pharmacokinetics (8). Pharmacodynamic interactions occur when foods interfere with the drug mechanism, while pharmacokinetic interactions affect its absorption, distribution, metabolism and excretion (ADME) system. Although not all FDIs are clinically relevant, some can have serious effects on the patient’s health, reducing the clinical efficacy of their treatment or increasing its toxicity (9). A well-known example is grapefruit juice, which affects drug metabolism by inhibiting enteric cytochrome P450 3A isoform and has more than 85 described FDIs (10). This includes a variety of drugs such as statins, immunosuppressants, corticosteroids, antihistamines or analgesics. On the other hand, FDIs can also improve drug access to the site of action, thus increasing its bioavailability, or prevent its toxic secondary effects (9). Therefore, knowledge of FDIs in clinical practice would be valuable to personalize dietary guidelines for each patient based on their medication regimen, in order to avoid harmful FDIs and exploit those that could be beneficial.

The broad variability in nutrition status, dietary habits, food composition and dietary supplement use, along with the widespread use of medicines, means that there is a vast amount of potential FDIs (11). However, knowledge of FDIs is scarce and mostly restricted to specific foodstuffs or bioactive compounds. European guidelines for the investigation of drug interactions during drug development only encourage FDI research when *in vivo* evidence suggests so or for specific products such as grapefruit juice (12). Pharmacovigilance is based on spontaneous reporting systems, where underreporting is common (13). Even within known FDIs, current knowledge of them in clinical practice has been reported to be unsatisfactory (14–16). The need for a better understanding of FDIs has been stated by the ELIXIR Community in Food and Nutrition (17) as well as by the 2020–30 Strategic Plan for NIH Nutrition Research, which includes a specific objective (Objective 4.1) to identify interactions between drugs, disease states and nutrition (18).

This need is compounded by the lack of focused resources for FDIs. FDI studies, while picking up traction in the last few years, are still not common and lag significantly behind drug–drug interaction (DDI) studies (2, 19). Efforts to organize information on interactions have focused on providing information on DDIs. Some examples are DrugBank (20, 21), drugs.com (https://www.drugs.com), medscape.com (https://www.medscape.com/), Rxisk.org (https://rxisk.org/), Rxlist.com (https://www.rxlist.com/) or Webmd.com (https://www.webmd.com), among others. These databases contain limited and incomplete annotations for FDIs with low overlapping between them, calling for better databases of known potential FDIs. Moreover, information on FDIs is mostly in the form of free text in scientific articles, knowledge bases not FDI-focused such as DrugBank (20, 21), case reports, clinical trials or even in patients’ discussion fora. The use of text mining to extract relevant information from unstructured data sources is an essential asset in these cases, and in the area of FDIs, it has led to the creation of semantic resources such as FIDEO (22), an ontology for FDIs that describes potential FDIs automatically extracted from scientific literature with more than 1 700 interactions integrated from online knowledge sources, POMELO (23), a manually annotated scientific corpus with information about FDIs from 639 Medline citations, corresponding to 5 752 sentences, or the Drug-Food Interaction (DFI) corpus (24), consisting of 2 271 abstracts of biomedical articles published by PubMed and 2 498 sentences that contain FDI and/or DDI information. However, these resources are rare and isolated, without direct applications for the end user, leaving room for improvements and for the development of new applications.

Other computational approaches such as physiology-based pharmacokinetic models, the identification of protein targets for a given food compound or bioactive, or the integration of high-throughput omics data might be useful to propose new putative FDIs for further *in vitro* or *in vivo* validation. The development of computational approaches based on molecular data that help the identification, extraction and organization of potential FDIs is needed to facilitate and accelerate the discovery and validation phases, reducing the time and cost of these studies. Moreover, the application of advanced omics techniques can help to better determine individual responses to food in health and disease (25, 26) while also allowing a deeper understanding of molecular mechanisms underlying FDIs (27, 28). Our approach towards this problem is based on molecular similarity, which allows to compare different molecular profiles and calculate measures to group samples or experiments with similar or divergent molecular profiles. Two profiles with different conditions are considered to be similar when there is a group of genes that are up- or down-regulated in the same direction in both conditions, i.e. upregulated in Conditions A and B or downregulated in both, while divergences occur when groups of genes are up- or down-regulated in opposite directions between both conditions, i.e. upregulated in Condition A and downregulated in Condition B or vice versa. This approach builds on the assumption that the effects that two given conditions produce at the transcriptomic level can help determine whether those conditions interact. Transcriptomic similarities have been used to compare gene and drug perturbations for drug prioritization (29) or to explore comorbidities (30, 31). In the context of FDIs, a similar strategy can be followed to compare the transcriptomic signatures of food compounds and drugs to infer potential FDIs.

Our work describes the computational strategies to identify and extract information about potential FDIs that were used to develop FooDrugs, a database created with the aim to organize information about potential FDIs from scientific documents and gene expression data while making it freely and easily accessible. Natural language processing (NLP) techniques were applied to identify and extract 1 108 429 potential FDIs for 50 960 foods and 161 809 drugs from the DDI corpus (32), PubMed (https://pubmed.ncbi.nlm.nih.gov/) and clinicaltrials.gov (https://clinicaltrials.gov/), while transcriptomic similarity profiling approaches were applied to identify 2 321 633 potential FDIs for 293 foods and 6395 drugs from gene expression profiles by combining data from the Gene Expression Omnibus (GEO) (33, 34) and the Broad Institute’s Connectivity Map (CMap) (35). This new database will give healthcare professionals (i.e. clinicians and nutritionists) better access to information about potential FDIs and will provide researchers in the area of nutrition, health and functional foods with a resource to evaluate new hypotheses and investigate the molecular mechanisms of FDIs.

## Materials and methods

### Extraction of potential FDIs via NLP techniques

#### Data collection

**Figure 1.**
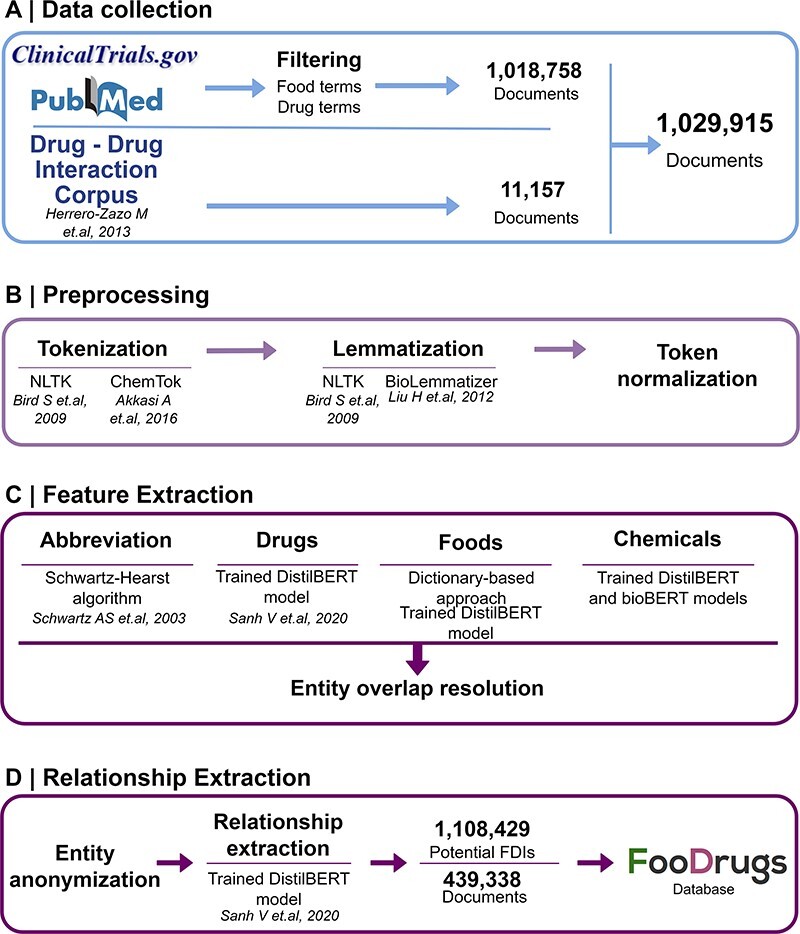
General overview of the NLP approach. (A) Data collection was performed by collecting documents containing at least one food and drug term in their titles and abstracts, as well as the whole DDI corpus. (B) Data preprocessing step is applied to the documents for better integration in the pipeline. (C) In the feature extraction step, different entities are recognized in the text via different methods, and entity overlap resolution is done when necessary. (D) Finally, for relationship extraction, entities are anonymized to work with the relationship extraction model used, and the resulting FDIs and documents are stored in the FooDrugs database.

Potential FDIs were mined from text documents using NLP techniques, as summarized in Figure 1.

The data collection method followed requires the generation of two lists of terms of foods in order to filter databases of documents. A list of food terms was generated by using different databases containing food compounds or bioactives and food descriptions. These databases include comprehensive databases such as FooDB (www.foodb.ca, 916 terms), a resource on food constituents, nutrients and bioactive compounds, or the Composition of Foods Integrated Dataset [(36), 946 terms], which stores data on the nutrient content of the UK food supply. Search terms were also obtained from more specific databases such as Phenol-Explorer [(37), 493 terms], which focuses on polyphenols present in food, the KEGG phytochemicals subset of KEGG BRITE (https://www.kegg.jp/brite/br08003, 2 882 terms) (38–40), PhytoHub (https://phytohub.eu/, 2 625 terms), which stores dietary phytochemicals and their metabolites, and terms annotated as food or food compounds in the DFI corpus [(24), 2 408 terms], the largest manually annotated corpus of biomedical articles published by PubMed for training models in DFI extraction. A full table of terms is available at Supplementary Table S1. The list of drug terms was collected from the Anatomical Therapeutic Chemical (ATC) classification system (https://www.whocc.no/, 5 863 terms), and terms annotated as drugs in the DFI corpus (5 500 terms). The whole collection of drug terms is shown in Supplementary Table S2.

Texts were obtained from different sources. First, texts with already annotated FDIs from the DDI corpus (32), a manually annotated gold standard corpus for DDIs containing 792 texts from DrugBank and other 233 Medline abstracts, were collected. Next, texts with potential DFIs were collected from PubMed (https://pubmed.ncbi.nlm.nih.gov/), a database of scientific articles, and from clinicaltrials.gov, a worldwide database of clinical studies (https://clinicaltrials.gov/). In order to collect the texts with potential FDIs from PubMed and clinicaltrials.gov, both lists of food and drug terms described earlier (Supplementary Tables S1 and S2) were used to select the documents that include at least one drug term and one food term in their description (i.e. title and abstract). To select these documents, a list of around 12 million texts containing food terms and a list of around 8 million texts containing drug terms were generated. Then, texts present in both lists were kept for further analyses.

#### Preprocessing

The Natural Language Toolkit (NLTK) (41) Python library was used to preprocess raw documents. NLTK is a leading platform for building Python programs to work with human language data. It provides easy-to-use interfaces to over 50 corpora and lexical resources along with a suite of text processing libraries for different steps, including tokenization and lemmatization of the documents. Tokenization allowed us to break text into individual units called tokens. Tokens are usually words or subword units like punctuation marks, numbers or symbols. A token normalization step was performed with the NLTK *Tokenizer* library, lowercasing the text, removing special characters and dividing strings into a list of substrings (tokens). Lemmatization allowed us to reduce words to their base or root forms (lemmas) while ensuring that the lemma belongs to the same language and makes sense in the context (e.g. lemmatization converts the plural noun ‘tomatoes’ to the singular form ‘tomato’). This process is useful for standardizing and normalizing text, making it easier to analyze and extract meaningful information. NLTK *Snowball and WordNetLemmatizer* libraries were used for this step. In addition, two domain-specific libraries were used: *ChemTok* to tokenize chemical names (42) and *BioLemmatizer* for the morphological analysis of biomedical literature (43).

#### Feature extraction

The tokenized texts were further processed by assigning part-of-speech tags to each token with NLTK. Named entity recognition (NER) was performed using several approaches, including classical methods based on word embeddings or gazetteers and deep learning methods based on neural networks.

First, the Schwartz–Hearst algorithm was applied to identify abbreviation definitions (44). Then, food NER was performed by a dictionary-based approach consisting of string matching with the food term lists in Supplementary Table S1 and a deep learning model based on the DistilBERT architechture (43). DistilBERT is a distilled version of the Bidirectional Encoder Representations from Transformers (BERT) model (45–47). BERT is a transformer-based deep learning technique that learns contextual relations (46, 47), while DistilBERT is a smaller and faster version that pre-trains a smaller representation model that can be fine-tuned afterwards (45). For entities with partial overlaps, only the longest term was considered. The next step was the detection of chemical compounds using two deep learning models based on BERT architectures trained for the identification of chemical compounds present in the text.

Drug NER was done by training a deep learning model with a transformer based on the DistilBERT architecture (45) in the DDI corpus (32). DistilBERT is a distilled version of the BERT model (45–47). BERT is a transformer-based deep learning technique that learns contextual relations (46, 47), while DistilBERT is a smaller and faster version that pre-trains a smaller representation model that can be fine-tuned afterwards (45). This corpus, along with some easy data augmentation techniques, was used to train a deep learning DistilBERT model, yielding a harmonic mean of the precision and recall (F1 score) of 0.88. After all NER methods had been applied, some entities were assigned to more than one category (food, chemical or drug), e.g. vitamin D. Partial overlaps were resolved by maintaining the longest entity. Then, if an entity had been marked as multiple types (e.g. food and drug), the class was assigned according to the following criteria: food > food chemical > drug > chemical.

#### Relationship extraction

All possible food–drug entity combinations were obtained for food–drug relationship extraction. Then, a deep learning model based on the DistilBERT architecture (45) was pretrained on the DDI corpus for FDI relationships (32). The DDI corpus is a manually annotated gold standard corpus consisting of 792 texts selected from the DrugBank database (20) and other 233 Medline abstracts. This fine-grained corpus has been annotated with a total of 18 502 pharmacological substances and 5028 DDIs, including both pharmacokinetic and pharmacodynamic interactions. Therefore, and assuming that the sentences used to describe DDIs will be similar to the ones describing FDIs, the DDI corpus is used to train a deep learning model to extract FDIs from documents. This model is entity agnostic, as entities are anonymized and only the context is relevant. This model had an F1 score of 0.77, obtaining a total of 1 108 429 potential FDIs from 439 338 text documents.

The performance of FooDrugs NLP method was assayed with the DFI corpus (24). The DFI corpus contains 2270 abstracts of biomedical articles accessible through PubMed and 2 498 sentences that contain DFI and/or DDI information. On this corpus, 1 001 abstracts contain a DFI key-sentence, and 1 269 do not include a DFI key-sentence. The FooDrugs NLP method was able to detect 4 998 interactions in 1 306 abstracts out of 2 270 (57.3%), 65% (654) of them in those annotated by DFI with key-sentence. In addition, an F1 score of 0.567 was obtained. These results are in concordance with the previous approaches based on BERT models in the DFI corpus, ranging from 49.4 to 55.0 (24).

### Inferring potential FDIs from gene expression data

**Figure 2.**
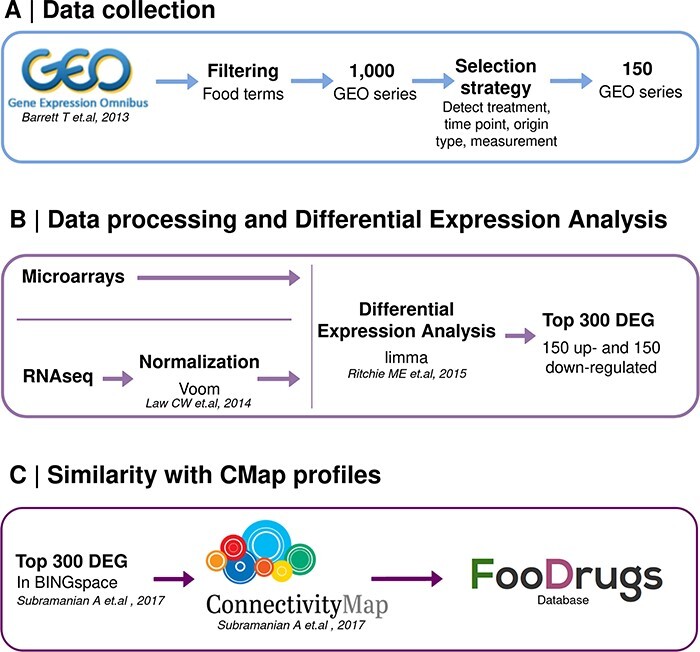
Schematic representation of the overall transcriptomic approach. (A) Data collection consists of the search of food transcriptomic studies with food keywords. (B) Data processing and differential expression analysis are performed with *limma* to get 150 up- and down-regulated genes in food condition vs control. (C) Genes present in BING space sent to CMap to compute similarity scores with drug transcriptomic profiles.

A second approach was designed to infer potential FDIs from the publicly available gene expression data, which quantifies the activity or level of expression of genes within a biological sample. It provides insights into which genes are actively being transcribed at a given moment in a cell, tissue or organism. A summary of this approach is shown in Figure 2.

### Data collection

Gene expression data were obtained from the GEO database (33, 34), a public functional genomics data repository. Each experiment on the platform includes information about the array or sequencing platform used (platform record), information about each sample characteristics, i.e. cell type, tissue, treatment or timepoint (sample records), and a series record that links together samples from the same submitter and provides a general description of the objectives of the study, together with other information like tables describing extracted data, summary conclusions or analyses. Each series record is assigned a unique and stable GEO accession number.

The list of food terms in Supplementary Table S1 was used to look for GEO studies testing food compounds, in addition to a collection of custom search terms that include ‘nutrient’, ‘nutrition’, ‘diet’, ‘natural products’, ‘phytochemical’, ‘nutrigenomics’, ‘bioactive and extract’, obtaining 1 000 studies. Later, these studies were filtered to avoid the word polysemy not related to food, e.g. transcription enrichment analysis, and keep those experiments referred to studies for a real food compound or bioactive effect.

An automatic selection strategy was defined to extract the treatment used in a study, the measurement of this treatment, the time point of each sample and the origin of sample, the information about the type of tissue essayed (cell line, tissue or primary cell) and the name of this cell sample (cell line name, tissue name or primary cell name). This strategy was implemented for all GEO studies collected in order to keep only studies for which the treatment compound was detected as a food compound or bioactive, and the origin of sample information was detected. The studies were discarded in the following cases: if the origin of sample or the treatment compound were not extracted and if the experiment did not contain two samples for controls and two samples for a given compound. A total of 150 studies were selected as potentially interesting and were therefore included in the FooDrugs database. For each study, the information stored in FooDrugs database is description (i.e. title and abstract), type of study, accession number, contributors, publication date and PubMed identifier. For each sample, the information stored is treatment compound, accession number, origin name and type, time point and concentration.

#### Data processing and differential expression analyses

Differentially expressed genes (DEGs), genes with changes in expression levels between two or more experimental conditions or sample groups, were identified comparing treated and untreated samples, defining a condition as a specific food/biocomponent tested at a particular concentration, time point and origin of sample within a study. A full table of RNA-seq and microarray studies in the FooDrugs database is available at Supplementary Table S3.

##### Microarray studies

The normalized data matrices for microarray studies were downloaded directly from GEO, and the *limma* R package (version 3.50.3) was used to determine the DEGs in each condition (48). Conditions in each study were defined by joining the variables *time, concentration, treatment and origin of sample* together. To obtain the DEGs in each experimental condition against the control, linear models were built within an empirical Bayes framework. Since each microarray tested around 40 000 probesets, adjusted *P*-values for multiple testing were calculated with the FDR via the Benjamini–Hochberg procedure. Afterwards, probesets were mapped to Entrez gene IDs using the platform annotations provided by GEO. Probesets that mapped to multiple genes were discarded, while genes that mapped to more than one probeset were represented only by the one with the largest average expression value. Average expression values were then ranked by adjusted *P*-values. From this ranking, the top 150 up- and down-regulated genes (having a logFC >0 or <0, respectively) were stored in FooDrugs, while the top 150 up- and down-regulated genes included in the CMap (35) were sent to this resource for further analyses, as described in the ‘Similarity with CMap profiles’ section.

##### RNA-seq studies

Gene count matrix files were downloaded from GEO for all RNA-seq studies. The *voom* method implemented in the *limma* R package was chosen for matrix data normalization, due to its good performance compared to other RNA-seq processing methods and because it fits directly into the *limma* pipeline (49, 50). Briefly, *voom* aims to compensate for the differences in sequencing depth and for the *count-variance* relation in the data, two properties of high-throughput transcriptomics data that difficult their normalization (49). Taking the log counts per million reads (log2cpm, logarithm in base 2) allows normalization for sequencing depth. It has been observed that larger counts have larger standard deviations. The log transformation counteracts this to some extent but overcompensates for small values. Then, smaller log2cpm counts may have higher variances, with technical variation decreasing with increasing log2cpm. To compensate for this effect, a gene-wise linear model that considers experimental design is fitted, generating a residual standard deviation per gene. Then, a lowess estimator is used to calculate the square root of the standard deviation with respect to the average log count for each gene. For each individual gene observation, based on its counts, its expected standard deviation is interpolated, and the inverse squared standard deviation for that observation is used as the weight for that observation log2cpm value in the *limma* pipeline described in the ‘Microarray studies’ section (49). Normalized data are then analyzed similarly to microarrays with *limma* to obtain DEGs. Then, average expression values are ranked by their adjusted *P*-value, and the top 150 up- and down-regulated genes are stored in FooDrugs database.

#### Similarity with CMap profiles

The top 150 up- and down-regulated genes obtained from the differential analysis pipeline with an Entrez ID, sorted according to *P*-value, were used to connect with the CMap from Broad Institute, according to the CMap guidelines (35). This resource houses over 1.5 million expression profiles with the transcriptional response to a large collection of small-molecule compounds as well as genetic reagents such as libraries of clustered regularly interspaced short palindromic repeats (CRISPR)/Cas9 constructs or short hairpin RNAs (35). These compounds and genetic constructs used to alter the cell transcriptome are referred to as ‘perturbagens’. One thousand hallmark transcripts are measured, from which it is claimed that around 81% of total transcripts can be inferred. The set of genes included in this database are called Best INFerred Genes (BING) feature space, a set of 978 landmark genes and 9 196 well-inferred genes. A more detailed explanation of the CMap tool and its data is given in the original manuscript by Subramanian *et al.* (35).

Thus, to build a CMap query, DEGs that were not in the BING space were filtered out. Then, the BING space DEGs were split into an overexpressed gene set and an underexpressed gene set according to their logFC. For gene sets with more than 150 genes, the top 150 were chosen based on their *P*-value. Experiments with <10 overexpressed or underexpressed genes were discarded.

The gene sets obtained from GEO food compound or bioactive transcriptomic experiments were used to query the CMap in order to determine gene similarity scores that could lead to potential interactions between each compound or bioactive (gene sets) and the drugs with expression profiles in CMap (35). Potential interactions are inferred by performing a Gene Set Enrichment Analysis (GSEA) on the overexpressed and underexpressed gene sets from the GEO experiment of interest. A GSEA is performed per query gene set to determine if it correlates positively or negatively with a given expression profile from CMap, obtaining two enrichment scores. Both enrichment scores are then used to calculate a *weighted connectivity score*, which is subsequently normalized per condition and cell type. Then, this score is compared to a reference database, finally obtaining the tau score, which ranges from −100 to +100. The sign of the tau score indicates the sign of the interaction, and its absolute value serves as a measure of its statistical significance. An absolute tau score of 90 indicates that 90% of the reference perturbations had a connectivity weaker than the query (Figure 2) (35). A more detailed description of the technical methodology is given by Subramanian *et al.* (35).

After performing the analysis, putative interactions are returned at various levels using a hierarchical data format. Results from *pert cell* annotation were selected to be included in FooDrugs database, meaning that the results correspond to a given perturbagen and cell line combination.

#### Binomial analysis of CMap compound labels

To perform binomial analysis on a list of CMap compounds, first a list with the relations of each CMap compound with its labels was generated or downloaded from CMap. Then, a binomial test was done followed by a Benjamini–Hochberg correction to get the over- and under-representation coefficients and their adjusted *P*-values for each of the CMap compound labels. This analysis was done using a custom script written in python2.

## Results

FooDrugs is a freely available database for potential FDIs composed of two sources of information: a text document component, where potential FDIs were extracted from a collection of texts from the scientific literature via NLP techniques, and a molecular-based component, where potential FDIs were inferred based on gene expression data. In total, the FooDrugs database contains 1 108 429 potential FDIs described in 439 338 scientific documents and 2 321 633 potential molecular interactions with an absolute tau score >90, integrating 3 923 GEO samples testing 462 different experimental conditions (defined as a specific food compound or bioactive tested at a particular concentration, time point and origin of sample) with 6 395 CMap perturbagens. The database is freely and publicly accessible at https://zenodo.org/records/8192515 (DOI: 10.5281/zenodo.6638469), under a Creative Commons Attribution 4.0 International License.

**Figure 3.**
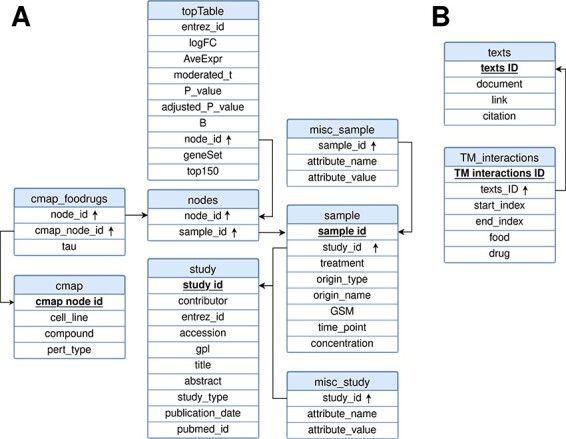
Relational model for the FooDrugs database. Primary keys for each table are marked in bold and underlined. Foreign keys are marked by an arrow pointing upwards. The database is formed by two independent components: (A) a molecular component, built from GEO studies involving food compounds or bioactives, and (B) a text mining component, built using NLP.

FooDrugs was built using MySQL (http://www.mysql.com) version 8.0.32-0. The relational model representing the database is shown in Figure 3. It is composed of 10 tables divided into 2 separate modules: the transcriptomic module, with 8 tables, and the text document module, with 2 tables. Tables in the text document module describe information about the texts in FooDrugs and all potential FDIs detected with the NLP pipeline. As for the transcriptomic module, tables ‘study’ and ‘sample’ describe information about the studies and samples retrieved from GEO, respectively. Information stored in the ‘study’ table includes the study description and citation information, while information stored in the ‘sample’ table contains the experimental condition used in each sample together with its accession number. Tables ‘misc_study’ and ‘misc_sample’ store additional information. The rest of the tables in the transcriptomic module are used to store the DEGs obtained using *limma*, as well as the gene sets that were sent to the CMap and information about their potential interactions with CMap transcriptomic profiles.

### Potential FDIs extracted from scientific documents

**Figure 4.**
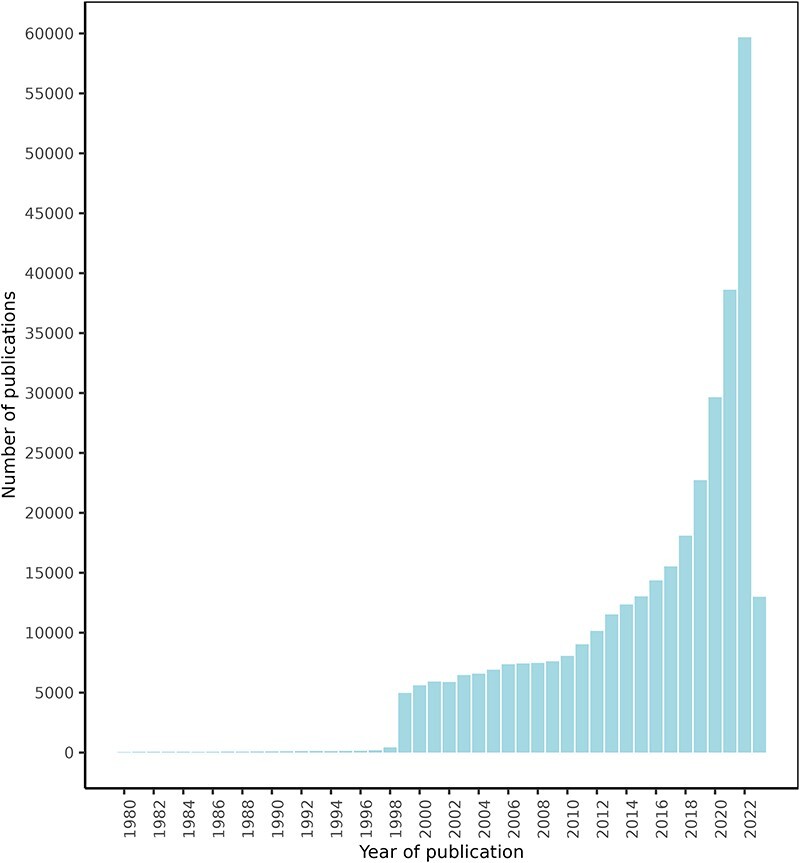
FooDrugs PubMed articles distribution by publication date. Articles with publication dates between 1980 and 2023 are shown. 779 articles published before 1980 are excluded from the figure. Search date was 28 July 2023.

After an initial search for texts mentioning a series of food and drug terms, which retrieved over 1 000 000 texts, the NLP approach explained in the ‘Materials and methods’ section retrieved a total of 1 108 429 FDIs from 439 338 texts. Of these texts, 425 023 were retrieved from PubMed, 13 778 from ClinicalTrials.gov, and 537 from the DDI corpus. Figure 4 shows the PubMed articles distribution by publication date. These documents describe potential FDIs between 50 960 food compounds or bioactives and 161 809 drugs, with 632 358 unique interactions between them.

### Food gene expression data

A total of 150 GEO series for human experiments were retrieved, including 29 RNA-seq and 121 microarray studies. Regarding sample origin, 86 experiments were performed in cell lines, 27 in primary cultures and 36 in biopsies, with 2 studies being performed in both cell lines and biopsies. A summary of the series characteristics is shown in Table 1. A full table with basic information of these series is available in Supplementary Table S3.

**Table 1.** Characteristics of the retrieved GEO series. First column displays the characteristics and the second column the number of studies following the characteristics

Characteristics	Number of studies following the characteristics
One-color arrays	110
Two-color arrays	11
RNA-seq	29
Number of different GEO platforms	63
Studies conducted on cell lines	86
Studies conducted on primary cultures	27
Studies conducted on biopsies	36
Series which study more than one compound	50
Series which study more than one concentration	102
Series which study more than one time point	38

Transcriptomic analyses were carried out on these series in order to identify DEGs between treated and untreated samples. Within each GEO series, a condition was defined as a food compound or bioactive tested at a specific time point, concentration and origin of sample. According to this definition, the database stores a total of 462 experiments for food compounds or bioactives. Each node contains information of an average of 2 451 DEGS (*P*-value < 0.05) ranging from 19 to 30 492 (median: 730).

### Potential FDIs inferred from transcriptomic profile similarity

A differential expression analysis was performed for each of the experiments in the database. Out of the 462 conditions in FooDrugs, 297 yielded upregulated and downregulated gene sets that could be used to query the CMap. As described in the ‘Materials and methods’ section, an interaction was inferred when both gene sets from the same condition correlated with a CMap expression profile in different directions, measuring its statistical significance by a tau score. This analysis allowed to retrieve 2 321 633 potential FDIs (absolute tau score > 90) with a total of 70 895 CMap profiles, which assayed 6 395 perturbagens from 5 different classes, including 2 545 drug compounds.

### Case studies

Two case studies are presented to demonstrate the usefulness of the FooDrugs database.

#### Looking for interactions with a specific food compound: resveratrol

A clear example for the exploitation of FooDrugs database is when a user is interested in the possible FDIs involving a particular food compound or bioactive without any previous assumptions or biological knowledge. A good example for this application might be a well-known compound such as resveratrol. This plant polyphenol is found in grape skin and seeds, and it has been shown to offer various health benefits, including antioxidative, anti-inflammatory and anticancer properties (51, 52). It may also play a positive role in autoimmune and inflammatory diseases, as well as cancer, diabetes or obesity (52). The potential health benefits of resveratrol and the interest to use it as a pharmaceutical drug make it crucial to be aware of potential drug interactions (51, 53). For instance, *in vitro* studies suggest that resveratrol might be a potential candidate against drug resistance in bladder cancer chemotherapy (54). However, resveratrol was also found to reduce the efficacy of paclitaxel both *in vitro* and *in vivo*, showing a possible contraindication (55). Instead of delving into scientific articles and databases to search for these specific examples, users can access the FooDrugs database to quickly and easily find potential FDIs through two different approaches:

- **NLP-based approach.** This approach returns potential mentions of FDIs in scientific texts, clinical trials and databases.
- **Transcriptomics-based approach.** The molecular component of the database can be used to search for an experimental setting testing resveratrol and compare it to the CMap expression profiles, inferring possible interactions from GEO-CMap edges with an absolute tau score >90.

First, the user could search for FDIs involving resveratrol that have been defined in the scientific literature. This returns 220 citations, with 154 unique potential FDIs. 198 of these citations come from PubMed articles, while the remaining 22 were found in clinicaltrials.gov. Many of these citations involve terms related to glucose metabolism and diabetes. For instance, ‘d-glucose’ and ‘insulin’ are two of the most repeated terms among the 220 citations. Other terms that can be found are ‘metformin’ or ‘hypoglycemic agents’, which are used to lower glucose levels in Type 2 diabetes mellitus patients, ‘sirtuin 1’, a protein involved in glucose metabolism and insulin sensitivity (56), and drugs to lower cholesterol levels such as ‘simvastatin’ or ‘HMG-CoA reductase inhibitors’. Moreover, other terms relevant to metabolism or metabolic disorders are found in this list, including ‘cholesterol’ or ‘triglycerides’ as well as the metabolic disorders ‘Type 2 diabetes mellitus’, ‘non-alcoholic fatty liver disease’ or ‘peripheral artery disease’.

Following the transcriptomics-based approach, the user’s questions would be: (i) which drugs could possibly interact with resveratrol and (ii) which changes resveratrol produces on gene expression patterns. Querying FooDrugs for GEO series where resveratrol is used as treatment returns nine different series, with seven experiments performed in one channel microarrays and two series with experiments performed using RNA-seq. For example, in the study with the GEO accession number GSE25412 (https://www.ncbi.nlm.nih.gov/geo/query/acc.cgi?acc=GSE25412), concentrations of 150 mM and 250 mM were tested on MCF7 cells after 48 hours of treatment, and nine samples are retrieved. The user can then find the DEGs related to a condition of interest, for instance, a resveratrol concentration of 150 mM. Querying FooDrugs database returns 28 significantly upregulated genes and 34 significantly downregulated genes. The result of querying the CMap with those DEGs included in the BING space retrieves potential interactions (i.e. with an absolute tau score >90) with a total of 2 779 CMap conditions, including 2 086 perturbagens: 607 drug compounds, 1 301 shRNAs and 871 cDNAs.

These results can be then used for further analyses. For instance, a binomial analysis on the mechanism of action labels for the 607 compounds that could potentially interact with resveratrol in the selected conditions reveals a significant overrepresentation of terms such as c-Jun N-terminal kinase inhibitors and cell cycle inhibitors (Benjamini–Hochberg adjusted *P*-value < 0.05), both of which have been described as candidates for anticancer therapies (57, 58). On the other hand, the significantly underrepresented terms include Src activators, CDC-like kinase (CLK) inhibitors, electrolyte reabsorption inhibitors and bile acid inhibitors. Interestingly, both Src and CLKs can have oncogenic roles, specifically in breast cancer; although through different mechanisms of action, Src is a tyrosine kinase, while CLKs are part of the splicing machinery (59, 60).

#### Looking for drug groups that interact with a specific compound: vitamin D

Fracture events are a major problem in elderly individuals and a serious public health problem that affects the quality of life of these people (61). Vitamin D, a fat-soluble vitamin essential for maintaining bone health, is a food supplement recommended to reduce the risk of fractures in elderly people (62); however, different trials have shown inconsistent results (63). Absorption of vitamin D from dietary sources is impeded in senior age due to various reasons, one of which might be the application of medications. A researcher interested in FooDrugs database information raised the assumption that different types of drugs can interrupt absorption of vitamin D in the gut, or consume it, as a derivative of cholesterol, causing vitamin D deficiency. Therefore, she was interested in a list of potential drug types that could deplete ingested or synthesized vitamin D, to enable interpreting patients’ vitamin D status results. This list will support the creation of a questionnaire for patients to further investigate the potential interaction of these drugs with vitamin D and its relationship with the possible loss of effectiveness of vitamin D in the prevention of fractures in elderly people.

First, a manual search in the literature by the researcher found a scientific revision (64) with 21 drugs classified by the authors as anti-inflammatory (9/21), antineoplastics (3/21), antiretroviral, antihypertensives, antibiotics and antiepileptics (2/21 each), and endocrine (1/21) drugs. In addition, a website (https://www.stlukes-stl.com/health-content/medicine/33/000724.htm) provided information for five potential drug categories interacting with vitamin D, being barbiturates, hydantoin derivatives, bile acid sequestrants, lubricant laxatives and histamine H2 antagonists. The FooDrugs database was used to test whether these lists of drug types were exhaustive and to verify the validity of the information found, by being able to track down the reference to the publications.

**Figure 5.**
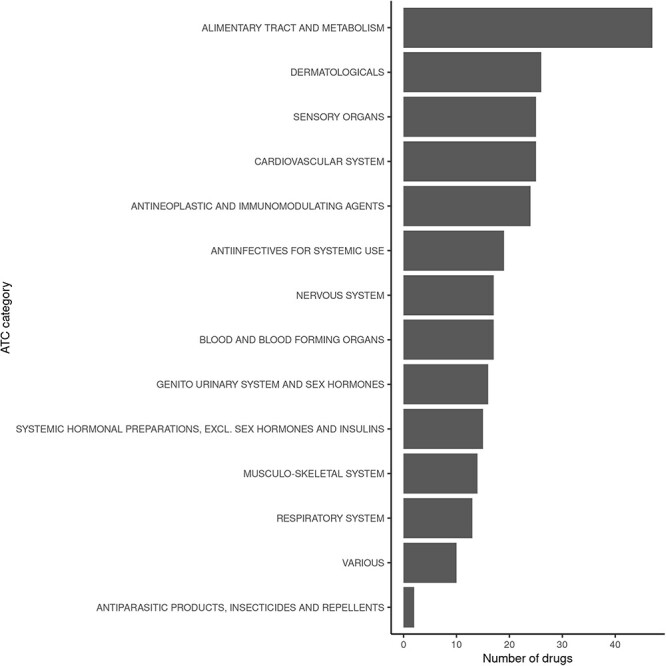
Classification of drugs found to interact with vitamin D in FooDrugs database, according to ATC classification. Only ATC Level 1 categories were considered for this comparison.

Querying FooDrugs for citations of possible vitamin D–drug interactions returns 878 texts from different sources, containing 1 146 interactions of vitamin D with 238 drugs. It might be interesting to assess whether the 238 drugs that were found to interact with vitamin D have similar properties or mechanisms of action. This was done by performing analyses with the ATC classification codes, which are based on therapeutic, pharmacological and chemical properties (https://www.who.int/tools/atc-ddd-toolkit/atc-classification). Among those drugs with potential interactions for vitamin D found in FooDrugs database, majority of them are implicated with the alimentary tract and metabolism ATC category, but many others are detected (Figure 5 and Supplementary Table S4). In conclusion, FooDrugs database has supported the work required by the researcher expanding the initial list of drug candidates and categories without complex and tedious manual search queries. In addition, FooDrugs database offers researchers access to the original publications to explore the potential mechanisms of degradation, if one is interested to know, which proves the traceability of the finding.

### Implementation of the FooDrugs web application

The FooDrugs database was implemented in a web application using a three-tier system architecture, i.e. a client–server software architecture that has three tiers or layers, namely the presentation, application and data tiers (65). The system is in a LAMP (Linux, Apache, MySQL and PHP/Perl/Python) environment (66). The data tier is linked to other external databases via PHP. The web application is available at http://imdeafoodcompubio.com/index.php/foodrugs/.

## Discussion

FDIs are a matter of concern in current clinical practice. With a greater consumption of dietary supplements and functional foods, coupled with a rise in non-communicable diseases and a growing number of polymedicated patients, the risk of adverse FDIs is at an increase (2, 4–6). Despite their relevance in clinical practice, the information regarding these interactions is scarce and scattered across the scientific literature, hampering its access by the research and the medical communities. FooDrugs database has joined the efforts to mine FDIs from the scientific literature with the development of an NLP workflow for FDI extraction. Additionally, the database also includes information for potential FDIs inferred from publicly available gene expression data. The result from combining these two approaches is an extensive database with a total of 318 133 unique potential FDIs, 142 885 from the molecular part and 175 248 from the text mining component.

The implementation of an automatic NLP pipeline has allowed the extraction of interactions from unstructured data sources, including scientific articles as well as clinical trials. Although more than 100 000 potential interactions have been obtained through this pipeline, it is important to note that these potential interactions collected do not encompass all possible drugs and foods. This limitation is a result of the method employed in the selection of relevant texts, based on lists of food and drug terms.

FDIs were also inferred by comparing gene expression profiles caused by food compounds or bioactives and drugs. Molecular similarity is an approach that allows to compare different molecular profiles and calculate measures to group samples or experiments with similar or divergent molecular profiles. FooDrugs molecular approach builds on the assumption that the effects that two given conditions produce at the transcriptomic level can help determine whether those conditions interact. With the same underlying hypothesis, the Connectivity Map has been used for purposes such as drug repositioning or the search for DDIs (67–70). In the context of drug repositioning, the aim is to find a drug with a transcriptomic effect opposite to that of the disease. This approach has been successful for repositioning the anticonvulsant topiramate in inflammatory bowel disease (67) and the antiulcer cimetidine in lung adenocarcinoma (68), as in both studies, the results were validated *in vivo*. DDIs have also been inferred from the CMap by performing GSEAs on every pair of drugs in the database. This was done to build the network in the Mode of Action by NeTwoRK Analysis tool (69, 70). Here, the principle of transcriptional similarity is used to build a network connecting CMap drugs to each other based on their expression profiles. Then, the authors identified communities in the network where most compounds shared a similar mechanism of action. Based on this work, the mechanism of action of nine anticancer compounds was predicted correctly (69, 70). The success of these studies supports the rationale behind the molecular component of the FooDrugs database, where an interaction between a food and a drug is assumed if their transcriptomic profiles are similar or different enough as measured by the tau score. This manuscript demonstrates how FooDrugs can be used to generate new hypotheses on the mechanism of action behind potential FDIs based on this reasoning. For instance, the case study involving resveratrol suggests that potential interactions with antineoplastic drugs might be more likely to happen through the disruption of processes such as cell cycle regulation rather than by altering RNA splicing.

Working in the context of FDIs implies a series of challenges intrinsic to these interactions: they might occur directly within the gastrointestinal lumen; foods can interact with drugs by indirectly affecting drug ADME (8, 9); and even dietary changes that affect the gut microbiota might alter drug pharmacokinetics (71). In addition to this, there are medications whose bioavailability or side effects change depending on whether they are administered together with any meal or on an empty stomach (8). Therefore, some FDIs might not be reflected at the transcriptional level and therefore would not be revealed by molecular similarity analyses, while it is also possible that not all interactions that can be inferred at the molecular level can happen in practice, or that they do, but by a different mechanism. In other cases, some FDIs have multiple underlying mechanisms (9), and thus, FooDrugs can help discover some of them. It should also be noted that, even if a real FDI is found, it might not be clinically significant (9). Keeping the complexity of this problem in mind, FooDrugs database can help researchers interested in the FDIs involving a specific bioactive compound, drug or a group of them, as demonstrated in the user case for vitamin D, to build reasoned hypotheses for their research or toeasily and quickly access data for further research.

## Conclusions

To our knowledge, FooDrugs is the first centralized database with textual and molecular information focused on potential FDIs. These complementary approaches provide the user with a wide range of information, from the transcriptional level up to the phenotypic level, without requiring any prior biological knowledge. This is achieved by gathering data from gene expression studies as well as information sourced from various scientific texts, including research articles and clinical trials.

FooDrugs provides users that are interested in the FDIs that involve a certain food compound or bioactive with: (i) texts that mention any drugs the compound might interact with; (ii) GEO studies where this compound was used as a treatment; (iii) how this treatment affects gene expression, by retrieving the DEGs compared with the control samples; (iv) the tau score for all the CMap chemical compounds after comparing the DEGs with their transcriptional effects; and finally, (v) the possible FDIs the compound is involved at the transcriptional level.

FooDrugs is a useful resource that perfectly complements the previous developments and will lead to an improvement of knowledge about FDIs, providing the basis to apply complex analytical approaches to deepen the understanding of the molecular mechanisms behind these interactions. Thus, this resource will help improve how FDIs are addressed from bench to bedside.

## Supplementary Material

baad075_Supp

## Data Availability

The FooDrugs database is fully available in Zenodo at https://zenodo.org/records/8192515, and can be accessed with DOI 10.5281/zenodo.6638469.
